# Prescription Behaviors of Primary Care Providers in Osteoporosis Management: A Survey

**DOI:** 10.7759/cureus.111213

**Published:** 2026-06-20

**Authors:** Vijayalakshmi Kumar

**Affiliations:** 1 Rheumatology, Raymond G. Murphy Department of Veterans Affairs Medical Center, Albuquerque, USA

**Keywords:** drug cost, prescription pattern, primary care setting, s: osteoporosis, survey analysis

## Abstract

Background

Osteoporotic fractures impose a substantial economic burden in the United States. Although multiple pharmacologic therapies reduce fracture risk, variation exists in prescribing patterns. Internal organizational data from our institution demonstrated disproportionate utilization of denosumab compared with zoledronic acid despite substantial cost differences. This study evaluated prescribing attitudes, cost awareness, and determinants influencing primary care providers’ osteoporosis medication selection.

Methods

A cross-sectional survey of primary care providers in New Mexico was conducted from April 18 to May 16, 2022. A convenience and snowball sampling strategy was used. Descriptive statistics, 95% confidence intervals, and chi-square testing were performed for subgroup analysis. An internal document review to determine relative acquisition costs of denosumab and zoledronic acid, and a limited literature review on this topic, were also conducted.

Results

Seventy-two providers responded (36% response rate). Although 75% reported prescribing denosumab more frequently, 48.6% incorrectly believed denosumab was similarly priced or less expensive than zoledronic acid. Only 26% correctly identified denosumab as much more expensive. Advanced practice clinicians underestimated costs more frequently than physicians (71% vs. 38%, χ²=6.36, p=0.012). Providers practicing >10 years also demonstrated significant underestimation (51.9%).

Conclusion

Primary care providers frequently underestimate the cost of denosumab despite reporting that cost influences prescribing decisions. Even long-practicing physicians demonstrated substantial misperception of drug pricing. Improved access to real-time cost information may promote more cost-effective osteoporosis management.

## Introduction

Osteoporotic fractures represent a major public health concern and are associated with substantial morbidity, mortality, and healthcare expenditures in the United States [1]. Osteoporosis is characterized by reduced bone mineral density, deterioration of bone microarchitecture, and increased susceptibility to fragility fractures [2,3]. As the population ages, the burden of osteoporosis-related fractures continues to rise, increasing the importance of cost-conscious and evidence-based treatment strategies.

Multiple pharmacologic therapies have demonstrated efficacy in reducing fracture risk, including bisphosphonates, denosumab, selective estrogen receptor modulators, and anabolic therapies [4-6]. Although anabolic agents may provide greater benefit in selected high-risk populations, antiresorptive therapies remain among the most commonly prescribed treatments in primary care settings because of familiarity, accessibility, and ease of administration [4-6]. However, substantial variation exists in the acquisition costs, dosing schedules, and healthcare expenditures associated with these therapies. Within our institution (Pharmacy and Therapeutics Committee, Osteoporosis Medication Cost Analysis 2022), internal utilization data demonstrated disproportionately greater use of denosumab compared with zoledronic acid despite substantial differences in acquisition cost . This observation raised concerns regarding whether provider perceptions regarding medication pricing aligned with actual institutional expenditures.

In routine clinical practice, prescribing decisions are influenced not only by efficacy and safety, but also by patient preference, convenience, insurance coverage, formulary restrictions, and physician perception of medication cost [7-15]. Prior literature has demonstrated that physicians frequently underestimate medication costs and that pharmaceutical promotion and direct-to-consumer advertising may indirectly influence prescribing behavior [9-15]. Despite this, relatively little literature has specifically evaluated cost awareness and prescribing attitudes related to osteoporosis medications among primary care providers.

The present study aimed to evaluate prescribing attitudes, determinants of osteoporosis medication selection, and provider awareness of relative drug costs among primary care clinicians in New Mexico. In addition, this study explored whether cost misperception differed according to professional role, years in practice, or practice setting. The novelty of this study lies in the integration of provider survey responses with internal institutional acquisition cost data to examine the relationship between prescribing perceptions and real-world healthcare expenditures.

## Materials and methods

Study design

This study employed a cross-sectional observational design using a structured electronic survey to evaluate prescribing attitudes and cost awareness among primary care providers. The survey component was supplemented by (1) an internal document review of medication acquisition costs and utilization patterns within a large integrated healthcare organization and (2) a focused literature review regarding physician cost awareness and determinants of prescribing behavior.

The study was conducted between April 18, 2022, and May 16, 2022.

Study population and setting

Eligible participants included primary care physicians [Doctor of Medicine (MD)/ Doctor of Osteopathic Medicine (DO)] and advanced practice clinicians (nurse practitioners and physician assistants) practicing in New Mexico who routinely prescribe osteoporosis medications.

Providers were excluded if: 1) They were specialists not typically managing osteoporosis (e.g., radiologists, pathologists). 2)They did not prescribe osteoporosis medications as part of routine clinical care.

The survey was distributed through a professional email distribution list. A convenience sampling approach was used, and respondents were encouraged to forward the survey to colleagues (snowball sampling). Based on distribution lists and outreach channels, approximately 200 providers were estimated to have received the survey.

Survey instrument development

The survey instrument was developed based on prior literature examining determinants of physician prescribing behavior [7]. The questionnaire assessed: 1) Demographic characteristics (sex, years in practice, practice type, professional role), 2) Sources of prescribing information, 3) Attitudes toward generic vs brand-name medications, 4) Perceived importance of efficacy, cost, and patient preference, 5) Perception of relative pricing between denosumab and zoledronic acid, and 6) Self-reported prescribing frequency.

To enhance internal validity and clarity, a pilot study was conducted among 15 physicians. Participants provided structured feedback regarding wording, interpretability, and survey length. Revisions were made to improve clarity and reduce ambiguity prior to final distribution.

The final survey consisted primarily of closed-ended questions with predefined response categories to facilitate quantitative analysis. The survey instrument used in this study is provided in the Appendix.

Internal acquisition cost review

To contextualize prescribing patterns, an internal review of the organization’s Value and Therapeutics Committee osteoporosis briefing document was performed . Data obtained included the following: we collected acquisition cost per unit of denosumab, annual per-patient cost based on dosing frequency, acquisition cost per unit of zoledronic acid, total institutional expenditure during the study period, and estimated patient cost-sharing (20% co-payment for Medicare beneficiaries). These internal data were used solely to quantify relative cost differences and were not linked to individual prescriber data.

Outcome measures

Cost perception responses were categorized into four predefined groups: “much more expensive,” “somewhat more expensive,” “about the same,” and “less expensive.” Responses of “about the same” or “less expensive” were classified as cost underestimation. Secondary outcomes included determinants of prescribing behavior, self-reported information sources, and subgroup differences according to professional role, years in practice, and practice setting.

Sample size justification

As this study was designed as an exploratory cross-sectional survey evaluating prescribing attitudes and cost perception among primary care providers, a formal a priori sample size calculation was not performed. The survey was distributed to approximately 200 eligible providers through professional distribution lists and snowball sampling methods. A final sample of 72 respondents was obtained. Based on an estimated sampling frame of approximately 200 eligible providers, the estimated response rate was 36%. Because survey distribution included snowball sampling, the true denominator could not be determined with certainty. The sample size was considered adequate for descriptive analyses and exploratory subgroup comparisons.

Statistical analysis

Descriptive statistics were used to summarize respondent characteristics and survey responses. Proportions were reported with 95% confidence intervals (CI) where appropriate. Cross-tabulation analyses were performed to evaluate subgroup differences in cost perception according to professional role (physician versus advanced practice clinician), years in practice (>10 years versus ≤10 years), and practice model (private practice versus managed care organization).

Chi-square (χ²) tests were used to assess statistical significance between categorical variables. Descriptive statistics, 95% confidence intervals, and chi-square analyses were performed using publicly available online statistical calculators provided by MedCalc (MedCalc Software Ltd, Ostend, Belgium). Degrees of freedom (df) were reported, and a two-tailed p-value <0.05 was considered statistically significant. All analyses were conducted at the aggregate level.

## Results

Response rate and demographics 

Participant demographics and response characteristics are summarized in Table 1. An estimated 200 primary care providers were reached, and 72 responses were obtained, yielding an estimated response rate of 36%. The majority of respondents were physicians (69.4%, 50/72), with advanced practice clinicians (APCs) comprising 29.2% (21/72). Nearly half of respondents (48.6%, 35/72) had been in practice for more than 20 years, and 72.2% (52/72) had more than 10 years of clinical experience. Most respondents (93.1%, 67/72) reported prescribing osteoporosis medications. 

**Table 1:** Demographic characteristics (N=72). Demographic characteristics of survey respondents. Data are presented as number (N) and percentage (%) of respondents. MD: Doctor of Medicine; DO: Doctor of Osteopathic Medicine; APC: advanced practice clinician.

Characteristic	N (%)
MD/DO	50 (69.4%)
APC	21 (29.2%)
>20 years practice	35 (48.6%)
Female	46 (63.9%)

Sources of information influencing prescribing decisions 

**Figure 1:**
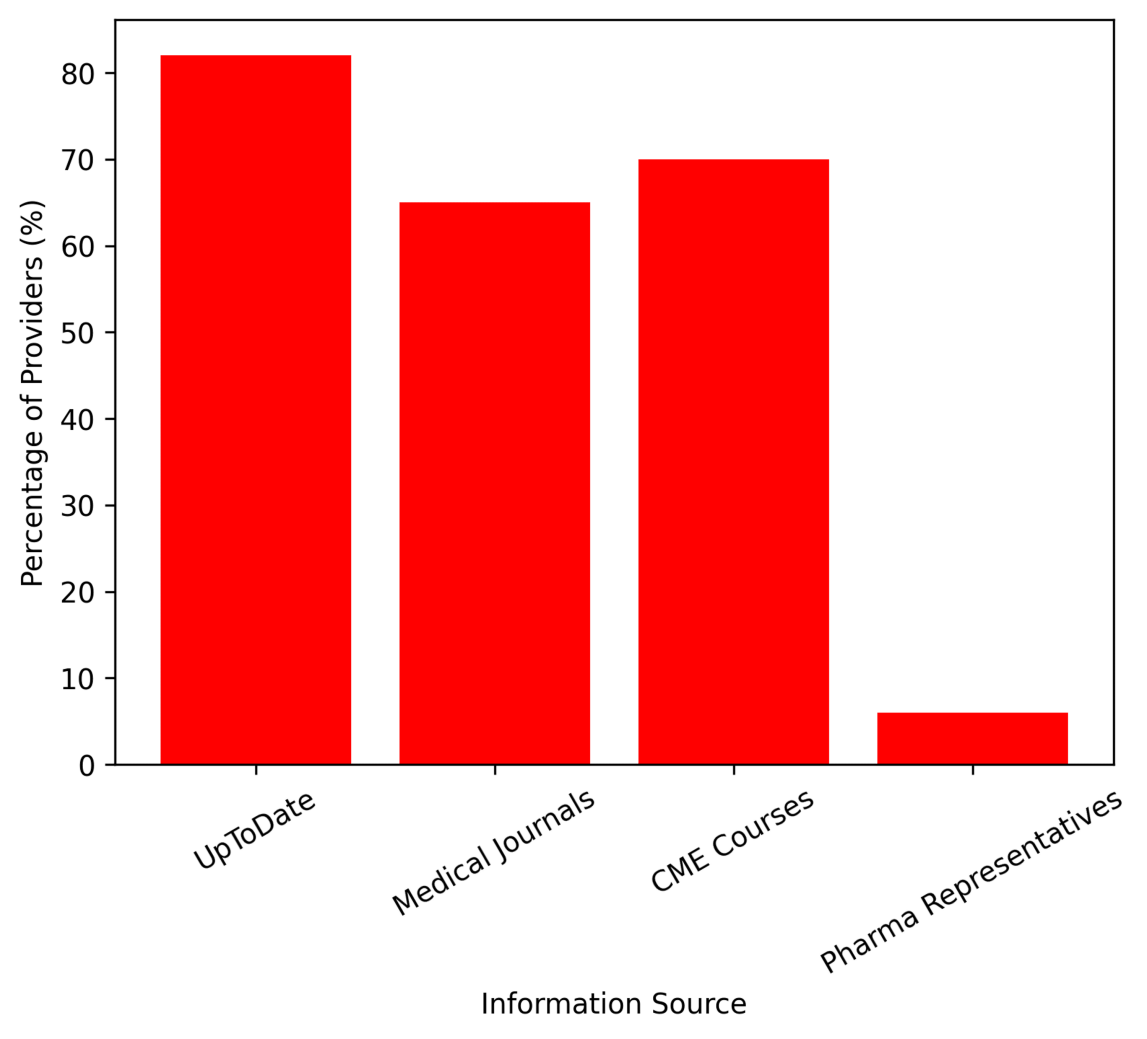
The primary information sources used by providers to guide prescribing decisions. Primary sources of information influencing osteoporosis prescribing decisions among primary care providers. Bar graph demonstrating the proportion of respondents reporting use of UpToDate, continuing medical education (CME) courses, medical journals, and pharmaceutical representatives as information sources influencing prescribing behavior. Data are presented as percentages of total respondents (N=72).

Sources influencing prescribing decisions are shown in Figure 1. UpToDate was the most frequently cited resource (approximately 80%+ of respondents), followed by continuing medical education (CME) courses (approximately 70%) and medical journals (approximately 65%). In contrast, only 5.6% (4/72; 95% CI 0.3-10.9%) reported pharmaceutical representatives as a source of prescribing information. This distribution suggests that providers perceive their prescribing decisions as primarily informed by academic or evidence-based resources. However, given the extensive literature demonstrating indirect pharmaceutical influence on prescribing behavior [9,10], the low self-reported reliance on pharmaceutical representatives may reflect social desirability bias rather than the absence of industry influence. 

Determinants of prescribing behaviors

Efficacy was identified as the most important factor in selecting osteoporosis therapy by 56.9% of respondents (41/72; 95% CI 45.5-68.4%). Cost to the patient was selected as the most important factor by 19.4% (14/72), while 16.7% (12/72) selected patient preference

Despite cost being identified as important by many providers, discrepancies emerged between the perceived importance of cost and the actual knowledge of medication acquisition costs.

Cost Perception and Underestimation of Denosumab Pricing

When asked to compare the price of denosumab with zoledronic acid, 48.6% (35/72; 95% CI 37.1-60.2%) believed denosumab was similarly priced or less expensive. Only 26% (19/72; 95% CI 16.2-36.6%) correctly identified denosumab as “much more expensive.” The remaining respondents believed it was only somewhat more expensive. Although respondents selecting “somewhat more expensive” correctly identified the direction of the cost difference, these responses may still underestimate the magnitude of the acquisition cost disparity between denosumab and zoledronic acid. Thus, nearly half of the providers significantly underestimated the relative acquisition cost of denosumab. Importantly, 75% (54/72) reported prescribing denosumab more frequently, and 67% indicated that patients preferred denosumab due to convenience (biannual subcutaneous administration). This suggests that prescribing preference may be influenced more by perceived convenience than cost considerations.

Subgroup Analysis of Cost Underestimation 

**Figure 2:**
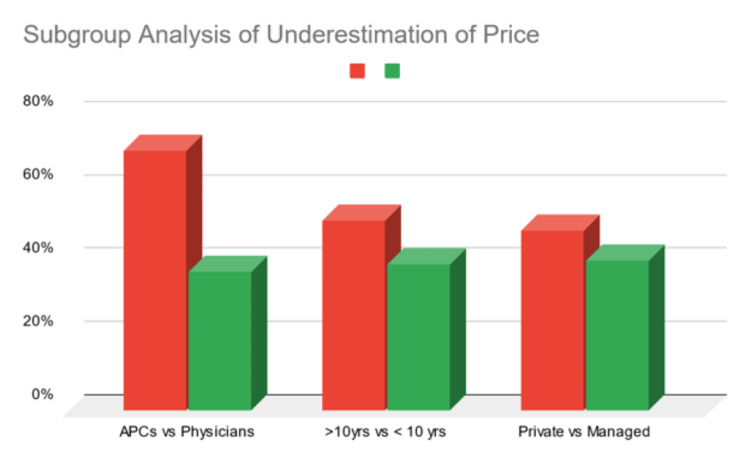
Subgroup analysis of denosumab cost underestimation. Subgroup analysis of denosumab cost underestimation among primary care providers. Bar graph comparing rates of cost underestimation between physicians and advanced practice clinicians (APCs), providers with >10 years versus ≤10 years in practice, and private practice versus managed care settings. Data are presented as percentages. Statistical comparisons were performed using chi-square testing. The difference between physicians and APCs was statistically significant (χ²=6.36, p=0.012), whereas differences according to years in practice (p=0.369) and practice model (p=0.586) were not statistically significant. Statistical significance was defined as p<0.05.

The subgroup analysis of cost underestimation is shown in Figure 2. 

Physicians vs APCs

Cost misperception was significantly more common among APCs (71%, 15/21; 95% CI 52.1-90.7%) compared to physicians (38%, 19/50; 95% CI 24.6-51.5%). This difference was statistically significant (χ²=6.36; df=1; p=0.012).

Years in Practice

Among providers with more than 10 years of practice, 51.9% (27/52) underestimated the cost of denosumab. Among those with fewer than 10 years of practice, 40% (8/20) underestimated the cost. Although this difference did not reach statistical significance (χ²=0.81; p=0.369), the data demonstrate that longer duration of practice did not confer improved cost awareness. Although years in practice were not significantly associated with cost underestimation, cost misperception was observed across all experience levels. 

Practice Model

Providers in private practice underestimated cost in 49% of cases compared with 42% among providers in managed care models. This difference was not statistically significant (χ²=0.29; p=0.586).

Acquisition cost comparison 

Acquisition cost comparison is shown in Table 2. An internal review of institutional acquisition data demonstrated that the acquisition cost of denosumab was approximately $1,126 per injection, corresponding to an estimated annual per-patient cost of approximately $2,250 with twice-yearly dosing. In comparison, the acquisition cost of zoledronic acid was approximately $45 for a once-yearly infusion. These findings indicated that denosumab was approximately 25-50 times more expensive than zoledronic acid within this healthcare system. These acquisition-cost estimates were derived from a single healthcare system and may not reflect pricing structures, negotiated contracts, reimbursement models, or formulary arrangements in other practice settings.” Given a 20% patient co-payment for many Medicare beneficiaries, denosumab may also impose substantially greater out-of-pocket costs for patients. The institutional expenditure data demonstrated disproportionate spending on denosumab relative to patient volume treated.

**Table 2:** Acquisition cost comparison. Institutional acquisition cost comparison between denosumab and zoledronic acid. Costs represent approximate acquisition costs obtained from internal organizational data. Annual denosumab costs reflect twice-yearly dosing. Data are presented as estimated cost per unit in US dollars.

Medication	Acquisition Cost per Unit	Notes
Denosumab injection	$1,126.00	Approx. $2,250/year: 25-50 x higher cost
Zoledronic acid	$45.00	Annual dosing: lower-cost alternative

## Discussion

This study demonstrates a substantial gap between the perceived importance of cost in prescribing decisions and actual knowledge of medication acquisition costs among primary care providers. Although 19.4% of respondents identified cost to the patient as the most important factor in selecting osteoporosis therapy, nearly half (48.6%) incorrectly believed that denosumab was similarly priced or less expensive than zoledronic acid. Only 26% correctly identified denosumab as “much more expensive.” Therefore, while approximately half of the respondents misclassified the relative cost relationship entirely, a larger proportion may have underestimated the magnitude of the cost difference. Cost underestimation was more common among APCs than physicians in this sample. However, given the relatively small APC subgroup and the inability to adjust for potential confounders such as practice setting, years in practice, formulary access, prescribing volume, and patient population, this finding should be considered exploratory and hypothesis-generating. 

However, the findings align with prior literature demonstrating that physicians frequently underestimate the cost of expensive medications while overestimating the cost of inexpensive ones. Allan et al. conducted a systematic review and found that physicians misestimated drug costs in the majority of studies examined, with a consistent pattern of underestimating high-cost drugs (binomial test, p<0.001) [10]. Our findings mirror this pattern.

Furthermore, although only 5.6% of providers in this study reported pharmaceutical representatives as a source of prescribing information, extensive evidence indicates that pharmaceutical promotion significantly influences prescribing behavior [8,9].

In addition, this study extends prior literature by demonstrating that cost misperception persists even among experienced clinicians. Providers practicing for more than 10 years underestimated the cost of denosumab in 51.9% of cases. Clinical experience alone does not appear to improve cost literacy. This suggests that systemic barriers to transparent pricing, rather than a lack of training, may lead to these persistent inaccuracies.

Pharmaceutical marketing expenditures in the United States exceed $60 billion annually [8]. Even when physicians report minimal reliance on industry representatives, interactions with pharmaceutical companies have been associated with increased prescribing of promoted medications [9,11]. Social desirability bias may contribute to underreporting of industry influence [12]. Direct-to-consumer (DTC) advertising may also shape patient-driven demand for medications [13,14]. DTC exposure increases patient inquiries and requests for specific medications [13], which may indirectly influence prescribing patterns. However, industry exposure, promotional contact, DTC advertising, patient requests, and formulary restrictions were not directly assessed in the present survey. These observations are provided as context from the existing literature and should not be interpreted as explanations for the prescribing patterns observed in this study. Importantly, this study did not evaluate the clinical appropriateness of individual prescribing decisions. Osteoporosis treatment selection depends on multiple patient-specific factors, including fracture risk, renal function, prior therapy, adherence considerations, contraindications, infusion access, and patient preference. From a comparative effectiveness standpoint, multiple pharmacologic agents reduce fracture risk [4,5]. Meta-analyses demonstrate that denosumab effectively reduces fractures and has a safety profile comparable to other therapies [6]. Certain therapies may demonstrate advantages over others in selected patients based on real-world clinical experience. Nevertheless, in primary care settings, when no clinically meaningful difference in efficacy or safety exists between therapeutic options, greater awareness of relative medication costs may help inform shared decision-making when clinically appropriate treatment options are available.

The internal acquisition data underscores the magnitude of cost discrepancy. Denosumab costs approximately $2,250 annually per patient compared with $45 annually for zoledronic acid - a 25-50-fold difference . Between June 2020 and June 2021, more than $1 million was spent on denosumab compared with less than $9,000 on zoledronic acid, despite only a threefold difference in patient volume. This represents a substantial difference in institutional expenditure relative to patient volume and may represent a potentially modifiable source of healthcare cost inflation.

These prescribing patterns also carry equity implications. Racial and socioeconomic disparities in osteoporosis screening and management have been documented [15,16]. Income deprivation has been associated with variations in anti-osteoporosis drug prescribing rates [17]. If higher-cost medications disproportionately burden patients through co-payments, inaccurate cost perception may inadvertently exacerbate inequities in access to care. Providing clinicians with real-time formulary and cost information has been shown to reduce medication expenditures. Tseng et al. demonstrated that access to formulary and cost data was associated with lower increases in yearly drug costs, averaging $208 per patient annually [18]. Extrapolated to osteoporosis management at scale, improved cost transparency may yield substantial savings.

Overall, this study suggests that cost misperception, convenience preference, and potential indirect marketing influences may collectively shape prescribing patterns in ways that increase healthcare expenditures without clear incremental benefit.

Statistical interpretation

The statistically significant difference in cost underestimation between advanced practice clinicians (71%) and physicians (38%) (χ²=6.36, p=0.012) suggests that professional role may influence cost awareness. However, the absence of statistically significant differences between providers with greater versus fewer years in practice (p=0.369) indicates that clinical experience does not independently improve the accuracy of drug cost estimation. Similarly, practice model (private vs managed care) was not associated with statistically significant differences (p=0.586), suggesting that organizational structure alone does not ensure improved cost knowledge. The response rate of 36% is consistent with published survey response rates among physicians and healthcare professionals [19]. However, the modest sample size limits power for subgroup analyses and may have contributed to type II error in comparisons of years in practice and the practice model. Despite these limitations, the magnitude of cost misperception (nearly half of respondents) suggests that the finding is unlikely to be attributable solely to sampling variability.

Strengths & limitations

Strengths include a combination of survey data and internal acquisition cost review, subgroup statistical analysis, real-world prescribing context, and direct linkage between perception and institutional expenditure. However, limitations include convenience sampling, modest sample size, and self-reported data, a single survey instrument [19,20]. An additional limitation is that the survey did not explicitly define cost as institutional acquisition cost, payer expenditure, or patient out-of-pocket expense. As a result, respondents may have interpreted the question differently, potentially contributing to misclassification of cost perceptions. Because snowball sampling was used, respondents may have differed systematically from nonrespondents with respect to cost awareness, prescribing patterns, practice setting, or interest in osteoporosis management. Therefore, prevalence estimates of cost misperception may not be fully generalizable to all primary care providers in New Mexico. It is also important to notice that clinical contraindications were not assessed [21]. The acquisition-cost data were obtained from a single healthcare system and may not be generalizable to other healthcare environments where medication pricing, payer contracts, reimbursement structures, and formulary arrangements differ. Nevertheless, the substantial cost differential observed between denosumab and zoledronic acid is consistent with published pricing data demonstrating higher acquisition costs for denosumab.” Patient preference may also contribute to increased utilization of denosumab. Prior studies demonstrate that women often prefer less frequent osteoporosis therapies, including weekly, monthly, or yearly regimens, over daily medications because of convenience and ease of adherence [22]. In addition, racial and socioeconomic disparities in osteoporosis evaluation and treatment have been previously documented and may further influence access to different therapeutic options [15,16,23]. Consumer attitudes toward direct-to-consumer pharmaceutical promotion may also contribute to patient-driven requests for specific therapies and indirectly shape prescribing patterns [13,14,24].

## Conclusions

Primary care providers demonstrated substantial inaccuracies in estimating the relative costs of osteoporosis therapies despite acknowledging the importance of cost in prescribing decisions. Nearly half of respondents underestimated the cost difference between denosumab and zoledronic acid, including many clinicians with extensive years of practice experience. These findings suggest that medication cost awareness remains limited even in experienced clinical settings and may contribute to prescribing patterns associated with increased healthcare expenditures.

Improving access to real-time formulary pricing information, increasing transparency regarding institutional medication costs, and promoting greater awareness of factors that influence prescribing decisions may help support more cost-conscious osteoporosis management. Greater integration of cost education into routine clinical practice may also improve value-based care while maintaining appropriate patient-centered treatment decisions.

## Appendices

**Table 3:** Survey instrument.

Question	Response Options
1. How long have you been in practice (post-residency/training)?	1–5 years; 6–10 years; 11–20 years; More than 20 years
2. In what setting do you provide MOST of your outpatient care? (Please select one)	Private single-specialty practice; Private multi-specialty practice; Group or staff model HMO or managed care organization; University/medical school-based practice; Veterans Affairs (VA); Community health center; Public health clinic; Other (please specify)
3. Do you practice in New Mexico?	Yes; No
4. Which one describes you?	MD/DO; Advanced Practice Clinician (APC); Other
5. What is your sex?	Male; Female
6. Do you prescribe osteoporosis medications?	Yes; No
7. When selecting a drug to treat osteoporosis, what is the most important criterion for you?	Efficacy; Route of administration; Cost to the patient; Patient preference
8. Which sources do you use to help with your prescription choices? (Select all that apply)	Medical journals; UpToDate; Textbooks; Pharmaceutical sales representatives; Continuing Medical Education (CME) courses; Other
9. How important is the cost of the drug when choosing a prescription?	Extremely important; Very important; Somewhat important; Not so important; Not at all important
10. In your opinion, how does the quality of generic drugs compare with brand-name drugs?	In general, generic drugs are higher quality than brand-name drugs; In general, generic drugs are lower quality than brand-name drugs; In general, generic drugs are the same quality as brand-name drugs; It depends on the specific drug
11. In your opinion, how does the safety of generic drugs compare with brand-name drugs?	Generic drugs are as safe as brand-name drugs; Generic drugs are safer than brand-name drugs; Generic drugs are less safe than brand-name drugs; It depends on the specific drug
12. Among the following two osteoporosis medications, which one do you prescribe more often?	Denosumab (Prolia®); Zoledronic acid infusion (Reclast®)
13. In your opinion, which of the following medications is more convenient for patients?	Denosumab (Prolia®) is more convenient; Zoledronic acid infusion (Reclast®) is more convenient
14. Based on your current understanding of the prices of denosumab injection and zoledronic acid infusion, please select the response you think is most accurate. (Please do not look this up.)	Denosumab is much less expensive than zoledronic acid infusion; Denosumab is somewhat less expensive than zoledronic acid infusion; The cost of denosumab is similar to zoledronic acid infusion; Denosumab is somewhat more expensive than zoledronic acid infusion; Denosumab is much more expensive than zoledronic acid infusion
